# The Psychometric Properties of the Positive and Negative Suicidal Ideation Scale among Portuguese Young Adults

**DOI:** 10.3390/ejihpe14040062

**Published:** 2024-04-04

**Authors:** Marta Brás, João Antunes, Cláudia Carmo

**Affiliations:** Psychology Research Centre (CIP), University of Algarve, 8005-139 Faro, Portugal; jpantunes@ualg.pt (J.A.); cgcarmo@ualg.pt (C.C.)

**Keywords:** suicidal ideation, psychometric properties, confirmatory factor analysis, reliability, validity

## Abstract

Preventing suicide has been a worldwide imperative for the last decade. Accurately assessing suicide risk is the first step towards prevention, and access to reliable tools that measure risk factors is essential to achieve this goal. The Positive And Negative Suicidal Ideation (PANSI) scale is a validated brief suicidal ideation scale that could prove useful to this goal due to its ability to measure both suicide risk and protective factors. The PANSI scale has been adapted to various languages and cultures across various clinical and non-clinical populations. Despite this, no Portuguese has been produced yet. The present study aimed to validate a Portuguese version of PANSI by evaluating its psychometric properties in a sample of 259 young adults. Confirmatory factor analysis showed that the PANSI showed good psychometric properties (TLI = 0.95), good reliability for positive ideation (α = 0.84), and excellent reliability for negative ideation (α = 0.96). The scale also showed good discriminative ability through prediction of a previous suicide attempt and good construct validity in both subscales. The Portuguese adaptation of the PANSI scale is a reliable measure of positive and negative suicidal ideation that could prove useful in both clinical and research settings.

## 1. Introduction

More than 700,000 people die due to suicide every year [1], and, more specifically, 9 out of 100,000 Portuguese individuals died to self-inflicted injuries in 2021, according to the latest data available [2]. A significant number of countries worldwide have made efforts to stop this problem from escalating through evidence-based practices. Accurately assessing suicide risk through validated measures and other indicators is essential for achieving this goal [3].

Suicide is preceded by various thoughts and behaviors, such suicidal and non-suicidal self-injury, suicide attempts, and suicidal ideation [4]. Suicidal ideation refers to any thoughts related to dying, spanning the entire range from occasional thoughts about death up to and including planning a suicide attempt [4], and is, therefore, a factor that is present throughout the entire suicidal process and is an important target for assessment. Recommended practice for suicide prevention clearly indicates that both risk and protective factors should be considered when assessing and intervening with potentially suicidal individuals [3]. Given the high prevalence of suicide worldwide, a brief measure that can assess these factors simultaneously is evidently useful both in identifying at-risk individuals or populations and in ensuring an evidence-based practice through the evaluation of potentially useful interventions. Although there are various measures to assess negative and positive suicidal ideation (reflecting the intent to die and to live, respectively), very few have considered both simultaneously [5,6].

To this effect, Osman et al. [6] developed the Positive and Negative Suicidal Ideation Inventory (PANSI). The PANSI is a strongly validated brief self-report measure of positive and negative thoughts related to suicidal behavior [6]. The original version was validated with university students [6] and has since been adapted for use among inpatient samples [7], high school students [8], and young adults [5]. It has also been validated adapted to various languages and countries, such as Colombia and Mexico [9,10], China [11,12], Pakistan [13], Malaysia [14], Nigeria [15], Spain [16], and Peru [17]. In all versions of this scale, a two-factor structure confirming the stated purpose emerged, both factors showed good internal consistency, and all 14 items of the original version were retained; more detailed information can be found in Table 1.

There is a recognizable need to produce age-stratified data in the field of suicidology [18], and suicide is particularly concerning among young adults, being the fourth leading cause of death in this population [1]. Obtaining stratified data on protective factors specifically can be particularly important in light of the fact that some of these factors are population specific, either in their presence or their absence [18]. It is also important to point out that the various protective and risk factors also interact with themselves and each other, necessitating an all-encompassing assessment to fully understand and intervene in any given population.

Despite the PANSI inventory having over ten adaptions in eight different countries, this measure is only available in five languages. Translating and adapting the PANSI scale into Portuguese, the ninth most spoken language in the world [19], specifically among young adults, can be a useful effort in combating suicide worldwide by expanding on the availability of a validated measure of positive and negative suicidal ideation. Additionally, the Portuguese population would benefit from access to this scale since, similar to the worldwide situation, despite there being negative (e.g., [20]) and positive (e.g., [21]) ideation scales, there is a similar scarcity for scales that measure both. As such, the present study establishes the present goals:

◦ To assess the internal structure of the PANSI inventory through confirmatory factor analysis (CFA).
◦ To examine the reliability of a Portuguese version of the PANSI inventory by assessing its internal consistency.
◦ To investigate the scale’s construct and discriminative validity.

Although the general goal of this study is to validate and investigate the psychometric of the PANSI scale, it is also important to further explain the subsequent goals. Firstly, CFA was chosen rather exploratory factor analysis (EFA), because the PANSI’s adaptations have been to consistently replicate its structure, particularly among adult populations. Additionally, investigating internal consistency is particularly important for the PANSI, seeing as one of its subscales, positive ideation, has shown issues with factoring into one single factor (e.g., Avendaño-Prieto et al. [16]).

## 2. Materials and Methods

### 2.1. Participants

Data 2343 collected through a snowball methodology by distributing an assessment battery on social medial platforms and asking participants to share them after completion. The only inclusion criteria were being between 18 and 30 years old and of Portuguese nationality, either from birth or by being a naturalized immigrant. The final sample contained 259 participants (M_age_ = 25.17; SD = 7.80), which was 78.4% female. In total, 30.9% of the sample had been diagnosed with a mental disorder in the past, and 43.6% was seeing or had seen a mental health professional in the past. In total, 61 participants (23.6%) had committed at least one act of self-injury without the intent to die, while 30 (11.6%) had made at least one suicide attempt in the past.

### 2.2. Materials

The Positive and Negative Suicidal Ideation Scale (PANSI) contains 14 items, measured on a 5-point Likert-type scale (1 = Completely Disagree; 5 = Completely Disagree). It contains 8 items that measure negative ideation and 6 that measure positive ideation, both showing strong internal consistency (α = 0.93 and 0.82, respectively) [6].

The Satisfaction with Life Scale (SWLS; Diener et al. [22], adapted to Portugal by Reppold et al. [23]) contains 5 items pertaining to overall satisfaction with life, measured in a 7-point Likert scale (1 = Completely Disagree; 7 = Completely Disagree). The original scale showed good consistency (α = 0.87), and its adaptation has acceptable consistency (α = 0.77). Previous validation studies of the PANSI have used this measure (e.g., [14]).

The Positive Affect Negative Affect Scale (PANAS; Watson et al. [24] adapted to the Portuguese population by Galinha and Pais–Ribeiro [25]) measures both positive and negative affect through 20 items, divided equally, rated on a five-point Likert scale (1 = Very Slightly; 5 = Extremely). The original scale and its adaptation showed good consistency for both positive (α = 0.88 and 0.86) and negative (α = 0.87 and 0.89) affect. This scale has seen use in previous adaptations of the PANSI (e.g., [5]), in part, due to evidence of its discriminant validity.

The Depression Anxiety Stress Scale-21 (DASS-21; Lovibond and Lovibond [26] adapted to the Portuguese population by Ribeiro et al. [27]) measures anxiety, depression, and stress through 21 items, distributed equally, scored on a 4-point Likert scale. This scale shows consistently good reliability and internal consistency, and has seen previous use in adaptations of the PANSI (e.g., [14]).

### 2.3. Translation and Data Collection Procedure

The translation process followed a backward–forward translation method to ensure the conceptual and cross-cultural equivalence of the content [28,29]. Forward translation into Portuguese was carried out by a native-speaking professor who was proficient in English. This version was backward translated into English by a psychologist who was fluent in both languages and had never worked with the used the original PANSI. Finally, a panel of four independent bilingual experts who were familiar with the instrument analyzed all three scales and made corrections to the Portuguese version based on any inconsistencies found between the original version and the back-translated English version.

Data were collected by personally distributing an access link to a form containing the aforementioned scales and a sociodemographic questionnaire to university students from University of Algarve. Additionally, the same access link was distributed through social media websites, using a snowball methodology.

### 2.4. Data Analysis

Before beginning data analysis, exclusion criteria were checked, leading to 27 participants being removed for being older than 30 years old. Since the form did not allow for blank answers, there was no need to correct for missing values. Correlational and internal consistency analyses were conducted using SPSS v.29.00.00, and AMOS v.29 was used for Confirmatory Factor Analysis (CFA). During CFA, estimation was calculated through maximum likelihood method complemented with bootstrapping (Bollen–Stine, 200 samples) to correct for multivariate non-normality (multivariate kurtosis = 207.26; critical ratio = 78.79), although univariate normality fell within the standard set by Kline [30]. Fit Indexes were evaluated in accordance with Kline [30] and Byrne [31]. Ideally, the absolute value of χ^2^/df will fall between 1 and 2, while the Tucker–Lewis Index (TLI) and Comparative Fit Index (CFI) are considered acceptable at 0.90 and excellent at 0.95 or higher. Root Mean Square Error of Approximation (RMSEA) and Standardized Root Mean Square Residual (SRMSR) should be lower than 0.05 and 0.08, respectively [31]. Average Variance Extracted followed Fornell and Larcker’s [32] threshold of >0.50. Logistic regression was used to study discriminant validity.

## 3. Results

### 3.1. Descriptive Analysis

Participants were mostly female (78.4%) (M_age_ = 25.17; SD = 7.80), Portuguese (96.1%), and single (86.9%). The majority of participants were students (49.0%), employed (30.5%), or both (13.1%), and 5.4% were unemployed. Regarding the population’s mental health, 30.9% had been diagnosed with at least one psychopathology in the past, 43.6% have been accompanied by a mental health professional in the past, 23.6% have committed at least one act of self-injury in the past, and 11.6% have made at least one suicide attempt. The male portion of the population (M_age_ = 26.43; SD = 9.73) was slightly older than the female portion (M_age_ = 24.82; SD = 7.15), although they reported lower rates of psychopathological diagnosis (21.4%) and professional support (32.1%) when compared to females (33.5% and 46.8%, respectively). Comparing suicide attempts within these groups, 12.3% of females and 8.9% of males had made at least one attempt. PANSI negative ideation scores were not significantly different between sexes (t = 0.64; *p* = 0.263), but positive ideation was significantly higher in males (t = −2.48; *p* = 0.007).

Among participants who did not engage in intentional self-injury (198), 79.3% had never been diagnosed with any psychopathology and 66.2% had never been accompanied by a mental health professional. Only four participants (2%) from this sample had made one suicide attempt. Inversely, from the 61 participants who had engaged in self-injury, 63.9% had at least one psychopathology diagnosis and 75.4% had been accompanied, while 42.6% had made at least one attempt to end their life. Participants who had made at least one suicide attempt were generally younger (M_age_ = 22.83; SD = 4.49), and, as expected, had higher rates of mental illness (70%), been accompanied by mental health professionals (73.3%), and engaging in self-injury (86.7%).

Rates of self-injury were also higher among participants with one prior diagnosis (48.8%), as were suicide attempts (21; 26.3%). A little more than half (57.5%) of the participants who had seen a mental health professional in the past had at least one prior psychopathology diagnosis, and 40.7% had engaged in self-injurious behavior, while 19.5% of them had made at least one suicide attempt up until that point.

Ordered mean scores for PANSI-negative suicidal ideation among the risk-factor subgroups were as follows: mental health care users (M = 16.29; SD = 10.16), individuals with a prior diagnosis (M = 18.38; SD = 11.00), those who engaged in self-harm (M = 22.25; SD = 10.91), and those who had attempted suicide (M = 25.97; SD = 11.50). For positive ideation, the scores were ranked as follows: those who had attempted suicide (M = 18.77; SD = 5.32), those who had engaged in self-harm (M = 19.32; SD = 5.18), individuals with a prior diagnosis (M = 20.05; SD = 5.07), and mental health care users (M = 20.46; SD = 4.87).

The mean score in the full sample for all study variables were as follows: PANSI-negative (M = 13.18; SD = 8.53), PANSI-positive (M = 22.16; SD = 4.57), PANAS-negative (M = 23.25; SD = 7.79), PANAS-positive (M = 33.56; SD = 7.80), DASS-21 (M = 43.30; SD = 15.57), and SWLS (M = 23.33; SD = 6.32).

### 3.2. Confirmatory Factor Analysis

Main fit index values for the two-factor model of the PANSI scale can be found in Table 2. Items 3 and 4’s error were set to be correlated in the model, as was the case for items 10 and 11, following the previous literature [14]. Both CFI and TLI were well within the set standards, particularly the CFI, which can be considered to be excellent. Although χ^2^/df was not within the ideal interval, it does fall within other, less restrictive standards, such as χ^2^/df < 3 (e.g., [33]). RMSEA also did not fall within the desired parameters, although it was close, which is important to point out since in structural equation modelling binary pass/fail decisions regarding a model’s validity are not as clear as in other fields of statistics [30]. Finally, SRMR was within the cut-off value for good fit.

### 3.3. Validity and Reliability Analysis

Standardized factor loadings and internal consistency metrics are presented in Table 3. Average Variance Extracted (AVE) for Negative Suicidal Ideation was 0.77 and for Positive Ideation it was 0.49. Negative Ideation showed excellent internal consistency (α = 0.96) and inter-item correlations varied between 0.57–0.88, while Positive Ideation showed good consistency (α = 0.84) and inter-item correlations varied between 0.35–0.66. Although internal correlations in the positive ideation subscale had some items above Kline’s [31] standard for assumption of non-collinearity (*r* ≤ 0.85), most of the items were under this threshold, and the ones that were not were only slightly above it, so it can be considered that there are no outstanding issues with collinearity in either subscale.

### 3.4. Construct Validity

Correlations between Negative and Positive Suicidal Ideation and all other measures are present in Table 4. All correlations were statistically significant. Positive ideation was positively correlated with both protective factors and negatively correlated with risk-factor measures, while the reverse was true for negative ideation. Positive and negative ideation were inversely correlated.

### 3.5. Discriminative Validity

Discriminative validity was assessed by testing each subscale’s ability to accurately predict one previous suicide attempt among the participants using binary logistic regression. As shown in Table 5, both subscales, independently, significantly predicted a previous suicide attempt in the expected direction.

Model fit tests also supported the discriminant validity of the positive subscale: Omnibus Coefficient Model (χ^2^ = 17.75; df = 1; *p* ≤ 0.001) and Hosmer/Lemeshow (Test: χ^2^ = 6.05; df = 6; *p* = 0.642). The same was true for negative ideation: Omnibus Coefficient Model (χ^2^ = 58.20; df = 1; *p* ≤ 0.001) and Hosmer/Lemeshow (Test: χ^2^ = 6.44; df = 4; *p* = 0.168). The overall classification accuracy for the PANSI-Negative was 92.8%, and for the PANSI-Positive it was 88.8%.

## 4. Discussion

The present study intended to assess the viability of the PANSI scale in a Portuguese population, expanding its access to a new language. During CFA, all fit indexes fell within the expected ranges, or came close to it, and the model fit resembled that of previous adaptations of the scale (e.g., [5,14]). These findings are evidence of the PANSI’s solid factorial structure. Based on these and previous results, it can be concluded that the PANSI can effectively differentiate two factors of suicidal ideation, indicating that the measurement of protective factors provided by this scale is not merely an absence of risk factors, being, in fact, its own construct.

This adaptation of the scale showed overall good internal consistency. Despite the fact that item 4 raised the subscale’s alpha, this increase was minimal, and the subscales generally showed similar or better internal consistency than other versions (e.g., [15,17]).

The scale’s construct validity was also similar to other adaptations, and showed that both positive and negative ideation effectively converge and diverge from other similar/opposite constructs, further evidencing the inventory’s ability to provide two distinct and opposite measures. Adaptations of the PANSI consistently used PANAS to investigate its construct validity, and those studies [5,7,8] showed similar subscale correlations. Although the only other adaptation to use the DASS-21 was [14], the present results corroborate the previous study. Finally, the SWLS correlation scores also replicate previous results [8,14]. As such, the Portuguese PANSI seems relate as expected to related constructs, such as anxiety, depression, affect, and satisfaction with life. These findings highlight the importance of measuring suicidal ideation directly and not simply assess it through related constructs.

When considering the scale’s discriminative validity, both ideations were able to effectively predict a previous suicide attempt, although negative ideation was a better predictor. These results are congruent with the analysis of discriminant validity in other psychometric studies of the PANSI [14], which also concluded that the higher the PANSI-negative score, the greater odds of a suicide attempt. This finding is crucial in proving that the PANSI does have practical utility, and can, therefore, be applied in clinical contexts. Future studies should expand on these findings by assessing whether the PANSI subscales are able to predict not just the presence of an attempt or the number of attempts, but also factors as frequency of self-harm or other behavioral and verbal signs of suicide risk [34].

Overall, the PANSI scale shows solid psychometric validity, even though negative suicidal ideation seems to show stronger properties. These results highlight a trend found in adaptations of the PANSI scale that generally indicate weaker psychometric properties in the positive ideation subscale of the positive ideation subscale of the inventory. Authors such as Chen et al. [12] point out that one possible reason for these results, particularly in non-clinical populations, might be that participants not fully understand, or perhaps might not have previously considered, the concepts associated with positive suicidal ideation. Yet, if this explanation was, by itself, sufficient to explain these findings, measures that assess similar constructs in similar conditions, such as the Portuguese version of the Reasons for Living Inventory in Young Adults [23], should have similar internal consistency scores, which is not the case. It is possible that one other reason for this discrepancy is due to the items themselves capturing positive suicidal ideation indirectly, through other con-structs. For example, item 2, “Felt confident about your ability to cope with most of the problems in your life?” might be tapping into self-efficacy as well as positive ideation, therefore explaining why it did not factor as well as the remaining items. Such a hypothesis highlights the difficulty of directly assessing positive suicidal ideation through a brief measure and should be pursued in future studies. Also, since positive and negative ideation are opposite constructs, some of the items might be running into the issue of double loadings. Although the presents results did not indicate that this was the case with the Portuguese PANSI, items such as “Felt that life was worth living?” show how this could be an issue.

## 5. Conclusions

This study was the first to investigate the PANSI in the Portuguese language. Additionally, this study also constitutes a valuable addition by studying young adults without limiting itself to university students. The Portuguese version of the PANSI seems to be a reliable brief measure of suicidal ideation. Implementing this tool into both research and clinical practice can be useful mainly due to its ease of application and its ability to measure the individual’s level of risk and protection, allowing for a full appraisal of suicide risk. The PANSI’s ability to accurately predict a future suicide attempt along with its relatively short application time are some of its key strengths and implementing it into set-tings, such as emergency rooms, could be particularly fruitful and ease the burden on these professionals. Exploring how healthcare professionals integrate both positive and negative suicidal ideation into interventions should be the focus of future research.

The PANSI is effective at measuring both positive and negative suicidal ideation, highlighting the important distinction between the absence of risk factors and the presence of protective factors. Findings relative to positive ideation’s weaker structure, both in this version and others (e.g., [14]), should be considered and addressed. The present study posited some possibilities to explain the current disparities between studies, but further research is needed. One possible avenue for such research could be the investigation of other constructs that could act as confounders, such as coping and self-efficacy.

It is important to point out that during CFA, the statistics that showed best fit were the ones that were least sensitive to sample size. The current study is limited by its relatively small sample, particularly in the context of psychometrics, so it is recommended that future studies attempt to replicate the present results with a larger sample. This study is also limited by the fact that more in-depth information on participants’ pre-suicidal behaviors was not collected, therefore not allowing for further analysis on the inventory’s relationship with other suicide-related constructs, beyond ideation, such as self-harm.

Future research should focus on expanding the PANSI to other age groups, particularly the elderly, or other sub-cultures within a given population, such as marginalized groups or minorities. Future studies should also go beyond considering previously assessed group differences, going beyond cultural and age-group differences, and testing, for example, the invariance of this inventory among sub-groups within the chosen population.

## Figures and Tables

**Table 1:** Summary of Published Studies Examining the Factorial Structure of the PANSI.

Authors	Country	Sample	N	Items	Factor Analysis	Factors	Cronbach’s α (NSI; PSI) ^1^
Osman et al. (1998) [6]	USA	University Students	450/286	20/14	EFA/CFA	2	0.91; 0.80/ 0.93; 0.82
Osman et al. (2002) [7]	USA	Adolescent Psychiatric Inpatients	195	14	CFA	2	0.96; 0.89
Osman et al. (2003) [8]	USA	Highschool Students	217	14	CFA	2	0.94; 0.81
Muehlenkamp et al. (2005) [5]	USA	University Students	398	14	CFA	2	>0.70
Villalobos-Galvis (2010) [9]	Colombia	Highschool and University Students	643	14	EFA	2	0.93; 0.84
Chang et al. (2009) [11]	China	Middle and Highschool Students	2341	14	EFA/CFA	2	0.94; 0.86
Yasien and Ahmad (2015) [13]	Pakistan	Adolescents	300	14	EFA	2	0.89; 0.73
Sinniah et al. (2015) [14]	Malaysia	Psychiatric Patients	483	14	CFA	2	0.93; 0.84
Aloba et al. (2017) [15]	Nigeria	University Students	530	14	EFA	2	0.76; 0.77
Avendaño-Prieto et al. (2018) [16]	Spain	Adolescent Students	1318	14	EFA	2	0.89; 0.77
Rodas-Vera et al. (2021) [17]	Peru	University	306/205	14	EFA/CFA	2	0.95; 0.85
Avendaño-Prieto et al. (2021) [10]	Colombia/Mexico	Adults (General Population)	815	14	CFA	2	--
Chen et al. (2021) [12]	China	Non-Clinical Adolescents	1198	14	CFA	2	0.92; 0.70

^1^ NSI = Negative Suicidal Ideation; PSI = Positive Suicidal Ideation.

**Table 2:** Fit indices for the Tested Two-Factor Model.

Fit-Index	Two-Factor Model
χ^2^	204.309
df	74
χ^2^/df	2.761 **
RMSEA (90% CI)	0.083 (0.069; 0.096)
SRMR	0.058
CFI	0.958
TLI	0.949

** *p* < 0.001.

**Table 3:** Standardized factor loadings and internal consistency metrics.

Item	Standardized Factor Loadings		Item Total Correlation		Alpha If Item Removed	
	Negative	Positive	Negative	Positive	Negative	Positive
2	--	0.53	--	0.48	--	0.84
6	--	0.73	--	0.66	--	0.81
8	--	0.70	--	0.64	--	0.82
12	--	0.63	--	0.58	--	0.83
13	--	0.73	--	0.63	--	0.82
14	--	0.84	--	0.76	--	0.79
1	0.86	--	0.86	--	0.96	--
3	0.93	--	0.92	--	0.95	--
4	0.66	--	0.66	--	0.97	--
5	0.85	--	0.83	--	0.96	--
7	0.92	--	0.90	--	0.96	--
9	0.94	--	0.92	--	0.95	--
10	0.89	--	0.88	--	0.96	--

**Table 4:** Correlation Between PANSI, PANAS (positive and negative), DASS-21, and SWLS.

	PANAS Positive	PANAS Negative	DASS-21	SWLS	Positive Ideation
Negative Ideation	−0.29 **	0.43 **	0.52 **	−0.35 **	−0.51 **
Positive Ideation	0.53 **	−0.37 **	−0.44 **	0.68 **	--

** *p* < 0.001.

**Table 5:** Results from logistic regressions.

Model	Predictor	Β	S.E.	Sig.	OR (95% CI)
1	PANSI- Positive	−0.18	0.043	≤0.001	0.840 (0.77; 0.91)
	Constant	1.60	0.858	0.063	4.94
2	PANSI- Negative	0.15	0.022	≤0.001	1.16 (1.11; 1.21)
	Constant	−4.72	0.538	≤0.001	0.009

## Data Availability

The data can be made available for consultation upon request from the corresponding author.
